# An integrated approach to epitope analysis I: Dimensional reduction, visualization and prediction of MHC binding using amino acid principal components and regression approaches

**DOI:** 10.1186/1745-7580-6-7

**Published:** 2010-11-02

**Authors:** Robert D Bremel, E Jane Homan

**Affiliations:** 1 ioGenetics LLC, 3591 Anderson Street, Madison, WI 53704, USA

## Abstract

**Background:**

Operation of the immune system is multivariate. Reduction of the dimensionality is essential to facilitate understanding of this complex biological system. One multi-dimensional facet of the immune system is the binding of epitopes to the MHC-I and MHC-II molecules by diverse populations of individuals. Prediction of such epitope binding is critical and several immunoinformatic strategies utilizing amino acid substitution matrices have been designed to develop predictive algorithms. Contemporaneously, computational and statistical tools have evolved to handle multivariate and megavariate analysis, but these have not been systematically deployed in prediction of MHC binding. Partial least squares analysis, principal component analysis, and associated regression techniques have become the norm in handling complex datasets in many fields. Over two decades ago Wold and colleagues showed that principal components of amino acids could be used to predict peptide binding to cellular receptors. We have applied this observation to the analysis of MHC binding, and to derivation of predictive methods applicable on a whole proteome scale.

**Results:**

We show that amino acid principal components and partial least squares approaches can be utilized to visualize the underlying physicochemical properties of the MHC binding domain by using commercially available software. We further show the application of amino acid principal components to develop both linear partial least squares and non-linear neural network regression prediction algorithms for MHC-I and MHC-II molecules. Several visualization options for the output aid in understanding the underlying physicochemical properties, enable confirmation of earlier work on the relative importance of certain peptide residues to MHC binding, and also provide new insights into differences among MHC molecules. We compared both the linear and non-linear MHC binding prediction tools to several predictive tools currently available on the Internet.

**Conclusions:**

As opposed to the highly constrained user-interaction paradigms of web-server approaches, local computational approaches enable interactive analysis and visualization of complex multidimensional data using robust mathematical tools. Our work shows that prediction tools such as these can be constructed on the widely available JMP^® ^platform, can operate in a spreadsheet environment on a desktop computer, and are capable of handling proteome-scale analysis with high throughput.

## Background

The utility of the multivariate statistical approaches to the analysis of peptide quantitative structure-activity relationships (QSAR) using partial least squares (PLS) was first demonstrated by Wold and colleagues in 1987 [1]. Since that time use of QSAR and PLS have become the norm in chemometrics and medicinal chemistry. QSAR methods have contributed greatly to developing an understanding of the physicochemical interactions between peptides and their receptors. Theoretical and practical aspects of these approaches have recently been discussed [2,3]. The role of peptide binding to MHC molecules was also first demonstrated in the 1970 s, but the QSAR approaches have not been widely adopted to understand MHC binding. Flower and colleagues have been pioneers in this area [4-6]. A recent publication by this group [7] is complementary to the work described here and in the accompanying paper [8]. Use of PLS for predicting subtle variations in peptide binding by MHC molecules has also been described by Tian *et al *[9,10].

Henikoff and Henikoff [11] laid the foundation for bioinformatics analysis of protein sequences and the concept of position-based sequence weighting as a means of analysis of protein sequence data. A wide array of MHC binding prediction schemes have evolved using position sensitive substitution matrices (PSSM) in combination with a number of machine learning approaches. The approaches have recently been reviewed [12-14]. The development of the field of immunological bioinformatics in general is described in Lund *et al *[15]. A variety of the different methods are publically available on web servers (Additional File 1; Table S1).

The practical limitations of bandwidth and computational power of web-based approaches quickly become apparent when attempting to analyze proteome-scale data from multiple strains of organisms. After experimentation with web-based systems and local versions of web-based applications, we found that recent versions of JMP^®^, a commercial software package for statistical analysis and data visualization http://www.jmp.com, had capabilities that made it possible to undertake proteomic scale analysis on a contemporary desktop computer. We demonstrate an alternative approach to MHC binding affinity predictions using a QSAR PLS approach and we compare the predictions to contemporary web-based prediction programs as benchmarks. We further demonstrate how this approach enables the visualization of several features of MHC binding interactions that have not been previously described. In an accompanying paper we show how QSAR PLS approach can be applied to visualize and quantify other aspects of MHC binding in a proteomic scale analysis [8].

We have used publically available datasets of amino acid physical properties and MHC-I and MHC-II binding for specific peptides to develop novel approaches for predicting MHC binding affinities. Principal component analysis of the amino acid physical property datasets was used to develop sets of z-scales, which were in turn used to convert the peptide sequences into vectors of z-scales. These vectors were then used to develop PLS and neural net (NN) models to predict the natural logarithm transformed binding affinity of the peptides.

Linear prediction equations of MHC binding were developed by standard PLS techniques. The prediction equations of the NN for each of the MHCs (essentially a non-linear PLS) were developed by two different methods in a multi-tour, random holdback process where different random training subsets were used to develop a final prediction. Assessment of over-fitting was statistically assessed in a systematic way as recommended by the JMP^® ^software. A unique feature made possible by the QSAR modelling approach is that the models were constructed having conceptual symmetries with the binding domains of the MHCs. The models were tested for reliability internally using standard statistical methodologies and then further validated by benchmarking them against prediction methods developed by others using identical peptide datasets. The benchmark comparisons consisted of two metrics: first, the r^2 ^of the relationship between experimental and predicted binding affinities was computed, which is most appropriate for continuous numerical data. Second, the AROC was computed after converting the continuous numerical predictions of the models to binary categories of "strong" binders and "weak" binders; a classification commonly used in evaluating other MHC binding. We further show that the symmetry between the statistical models and the MHC binding domains makes it possible to visualize aspects of the physicochemical properties of the binding reaction between the peptide and the MHC molecule.

## Results and Discussion

### Amino Acid Physical Properties

**Figure 1:**
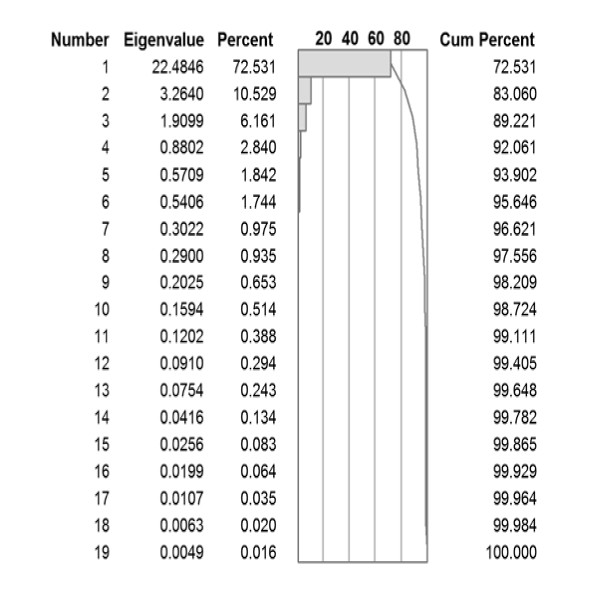
**Principal component analysis of 31 different studies estimating different physical properties of amino acids**.

Source data for the amino acid physicochemical properties were obtained from the proteomics resource at the Swiss Institute for Bioinformatics. Attempts were made to balance the types of physical data used and to obtain independent measurements of related physicochemical properties from two or more different studies. A total of 31 sets of physical parameters were tabulated (Additional File 2; Table S2) and the principal components (PC) computed from the correlation matrix. Results of this analysis are shown in Figure 1. The first three principal components account for approximately 90 percent of the variance in the 31 datasets. Table 1 is a listing of the 20 amino acids found in proteins, sorted independently by each of the first three principal components. It is apparent that the first PC is correlated with the polarity or hydrophobicity of the amino acid, and the second PC with molecular size. The underlying physical correlate of the third principal component is less obvious, but is generally considered to be electronic in nature [15]. The two sulfur-containing amino acids (cysteine and methionine) and histidine, with an imidazole ring, are ranked at one end of the third PC. Other amino acids with aromatic rings are ranked close behind. The actual z-scale numbers are slightly different from those originally reported by Wold *et al *[1,16], likely because we used a significantly larger dataset to develop the PC.

**Table 1:** First three principal components of amino acid physical properties.

Amino acid	Principal Component 1	Amino acid	Principal Component 2	Amino acid	Principal Component 3
**K**	-6.68	**W**	-3.50	**C**	-3.84
**R**	-6.30	**R**	-2.93	**H**	-1.94
**D**	-6.04	**Y**	-2.06	**M**	-1.46
**E**	-5.70	**F**	-1.53	**E**	-1.46
**N**	-4.35	**K**	-1.32	**R**	-0.91
**Q**	-3.97	**H**	-1.00	**V**	-0.35
**S**	-2.65	**Q**	-0.47	**D**	-0.18
**H**	-2.55	**M**	-0.43	**I**	0.04
**T**	-1.42	**P**	-0.36	**F**	0.05
**G**	-0.76	**L**	-0.20	**Q**	0.15
**P**	-0.03	**D**	0.03	**W**	0.16
**A**	0.72	**N**	0.21	**N**	0.30
**C**	2.11	**I**	0.29	**Y**	0.37
**Y**	2.58	**E**	0.34	**T**	0.94
**M**	4.14	**T**	0.80	**K**	1.16
**V**	4.79	**S**	1.84	**L**	1.17
**W**	5.68	**V**	1.98	**G**	1.21
**L**	6.59	**A**	2.48	**S**	1.30
**I**	6.65	**C**	2.74	**A**	1.42
**F**	7.18	**G**	3.08	**P**	1.87

Principal component 1 is correlated with polarity or hydrophobicity, principal component 2 is correlated with size. The physical correlate of principal component 3 is not obvious but is generally considered to be electronic.

PC are mutually orthogonal and are effectively uncorrelated proxies that embody the effect of the physical properties of amino acids found in proteins. An additional property characteristic of PC is that they are numerically appropriately weighted for their relative contributions to fitting a multivariate regression.

### Human MHC-I and MHC-II Datasets

**Figure 2:**
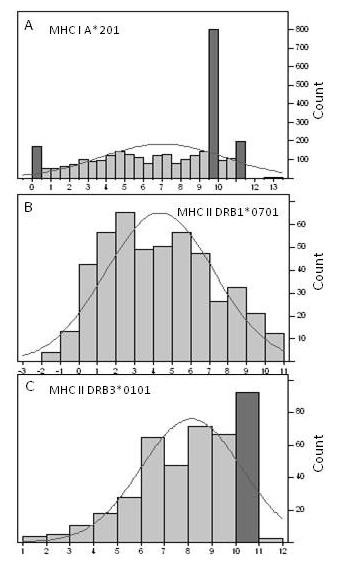
**Representative distributional properties of ln(ic50) binding data in the MHC-I and MHC-II benchmark data sets**. (A) MHC-I A201, (B) MHC-II DRB1*0701 and (C) DRB3*0101. The dark bars in the histogram contain a preponderance of identical ic50 binding measurements. The curve is a normal distribution fit to the particular dataset. x axis = ln(ic50). Count is the number of peptides in the particular bin of the histogram.

Extensive datasets of binding affinities of synthetic peptides have recently been made publicly available at the Immune Epitope Database (IEDB) [17-19]. Because we wanted to use the web-based programs NetMHCII and NetMHCIIPan at the Center for Biological Sequence Analysis (CBS) as comparators, we downloaded MHC-I and MHC-II databases from CBS [20]. Although the principles can readily be expanded to peptides of other sizes, the work described herein is restricted to 9-mers for MHC-I and 15-mers for MHC-II. The datasets downloaded contained binding affinity measurements as ic50 values for 29,336 9-mers for 35 alleles of MHC-I and 9,117 15-mers for 14 alleles of MHC-II (Additional File 3; Table S3a. and Additional File 4; Table S3b.). The binding data were natural logarithm-transformed ln(ic50) and the distributional characteristics of the binding data examined. The paper that is the primary source of the MHC-II dataset does not describe the experimental strategy for choosing the various peptides [19] but clearly the experimentalists designed the peptides in the datasets based on prior knowledge of binding characteristics. Several features of the datasets were noted. All of the datasets consist of several subsets. For example with the MHC-II allele datasets there are three major subsets: 232 peptides (232-subset) have been tested in all 14 HLA MHC-II supertypes, 167 (167-subset) with 11/14 supetypes, and 49 with 8/14 supertypes. In addition to these larger subsets there are subsets with fewer peptides that have been tested in various combinations of alleles and some of these have quite high average affinities. For example, one subset has an e^4.1 ^higher average affinity than the 232-subset. The amino acid combinatorials are different among the different subsets and they have statististically different means and variances. The experimentally measured average binding affinities of the 167 peptide subset is consistently greater than the 232 peptide subset in every one of the alleles where both have been tested. The difference between the means of the 232-subset and the 167-subset is e^2.2 ^(167-subset has statistically significant higher affinities) and among the different alleles the ratios of the means range from about 3-fold to 60-fold. A second feature apparent in the datasets is that the frequency distributions of the datasets have some anomalies; several are shown in Figure 2. The three highlighted bars in the histogram in Panel 2a. each contain a large fraction of the data points all ascribed exactly the same number 124/166 (1 nM), 701/800 (20,000 nM) and 168/195 (78,125 nM). Measurement of low affinity binding, where very small fractions of the total ligand are bound, are sometimes given identical scores. This is the assay experimental limit. Wang *et al *[19] do not indicate the source and history of the datasets; it is possible that they are composites of measurements made at different times and under different laboratory conditions. The reason for the cluster of high affinity binders is likewise unclear, but is also likely to represent an experimental limitation. Wang *et al *[19] state that they capped the binding affinities at 50,000 nM which is at odds with what is found in the datasets. Panels 2b. and 2c. are from the smaller MHC-II datasets; these have fewer anomalies than seen in Panel 2a. Clearly peptide synthesis was done by systematically varying different positions in the peptides, a process which likely gave rise to the similarities described by El-Manzalawy *et al *[14], who systematically evaluated the effects of similarity reduction on a number of commonly used datasets and concluded that regardless of the method employed, the performance of classifiers was substantially below that reported.

### Use of Principal Components of Amino Acids in PLS Analysis of MHC Binding

Peptides of 9-mers and 15-mers were converted into 27 (9 × 3) and 45 (15 × 3) vectors of principal components. This type of descriptor is commonly used in QSAR analysis where it is known as the "z"-scale [3,21]. These z-scales were used in a partial least squares (PLS) analysis of the binding data. Each amino acid in a 9-mer is replaced by three z-scale descriptors. {z_1_(aa_1_), z_2_(aa_1_), z_3_(aa_1_)}, {z_1_(aa_2_), z_2_(aa_2_), z_3_(aa_2_)}... {z_1_(aa_9_), z_2_(aa_9_), z_3_(aa_9_)}. A 15-mer for MHC-II analysis has a correspondingly larger set of descriptors.

### PLS Binding Affinity Predictions

The z-scale descriptors were used in PLS to develop prediction equations of peptide binding by MHC-I and MHC-II. This is essentially the approach pioneered by Hellberg *et al *[22], which was the genesis of the use of QSAR techniques for peptide binding. For evaluation of multivariate datasets such as these, the variable importance projection (VIP) is a very useful method to reduce the dimensionality of the data and to produce a single metric that ranks the relative importance of a particular predictor in the overall response (2,3). The VIP is an output generated using all the experimental measurements in the MHC datasets (29,336 MHC-I and 9,117 MHC-II) and the relationships among them. Any VIP metric with a value greater than 1 is considered to be of importance in the overall prediction. A by-product of the symmetry between the statistical models for the MHC binding interaction and the underlying binding reaction itself is that it possible to visualize and conceptualize the physicochemical properties of the interaction. The matrices of VIP values for all the alleles of both sets of HLA molecules are found in Additional File 5; Tables S4a and Additional File 6; Table S4b. The PLS models for all of the MHC-II alleles show evidence of significant latent factors, with twelve of the alleles having evidence of two latent factors and two of the alleles having three [2,3,23]. Through experimentation (data not shown) it appeared that the latent factors arise as a consequence of the clustering of different subsets having different means as discussed above. It is expected that the least squares processes of PLS should be less sensitive to the anomalies in the datasets than PSSM based methods.

MHC binding predictors have traditionally used categorical classifiers with motifs or PSSM (see Table 2 of Wang *et al*) [19]. Using a performance metric of area under the receiver operator curve (AROC), a strong binder is considered to be one with an affinity <= 50 nM, a weak binder >50 < = 500 nM, and those over 500 nM are considered non-binders [15]. As seen in Table 2, PLS performs most comparably to NetMHCIIPan in its categorical classification as strong or weak binders. For regression analysis of continuous data the application of a categorical metric is not appropriate. However, to make it possible to compare our results with prior published data we performed standard regression analysis of continuous data and computed an r for that fit between the experimental binding data and the predictions and then also transformed the output into the conventional binding affinity categories to produce an AROC metric. The results are shown in Table 2 and graphical visualization of the VIP is described below.

**Table 2:** Comparison of Partial Least Squares and Neural Net.

	PLS			Method 1			NetMHCII			NetMHCIIPan		
	**AROC**		**r^2^**	**AROC**		**r^2^**	**AROC**		**r^2^**	**AROC**		**r^2^**
	**SB**	**WB**		**SB**	**WB**		**SB**	**WB**		**SB**	**WB**	

DRB1*0101	0.713	0.579	0.541	0.838	0.645	0.796	0.848	0.691	0.811	0.835	0.647	0.753
DRB1*0301	0.675	0.610	0.476	0.987	0.954	0.996	0.958	0.882	0.966	0.841	0.602	0.736
DRB1*0401	0.690	0.537	0.491	0.986	0.956	0.995	0.951	0.845	0.945	0.778	0.631	0.636
DRB1*0404	0.695	0.559	0.595	0.986	0.961	0.995	0.940	0.845	0.954	0.854	0.630	0.769
DRB1*0405	0.702	0.577	0.527	0.985	0.966	0.996	0.927	0.846	0.947	0.809	0.588	0.682
DRB1*0701	0.729	0.612	0.559	0.987	0.958	0.997	0.965	0.893	0.963	0.879	0.716	0.801
DRB1*0802	0.776	0.602	0.587	0.990	0.980	0.997	0.979	0.880	0.973	0.841	0.550	0.770
DRB1*0901	0.659	0.532	0.403	0.988	0.961	0.997	0.969	0.899	0.956	0.813	0.576	0.673
DRB1*1101	0.681	0.565	0.550	0.981	0.957	0.996	0.968	0.893	0.969	0.855	0.594	0.787
DRB1*1302	0.600	0.521	0.441	0.978	0.830	0.997	0.981	0.837	0.965	0.806	0.579	0.759
DRB1*1501	0.656	0.552	0.494	0.987	0.960	0.995	0.940	0.795	0.945	0.768	0.544	0.667
DRB3*0101	0.595	0.510	0.451	0.983	0.932	0.996	0.956	0.872	0.935	0.879	0.613	0.737
DRB4*0101	0.724	0.667	0.604	0.987	0.966	0.997	0.686	0.942	0.976	0.892	0.621	0.795
DRB5*0101	0.727	0.607	0.553	0.985	0.958	0.997	0.960	0.884	0.965	0.872	0.649	0.789

**Average**	**0.687**	**0.574**	**0.519**	**0.975**	**0.927**	**0.982**	**0.931**	**0.857**	**0.948**	**0.837**	**0.610**	**0.740**

The performance of partial least squares (PLS) compared to the neural network regression base on amino acid principal components (NN PCAA) described with two neural network predictors based on substitution matrices. SB and WB columns are the area under the receiver operator curve (AROC) obtained by converting the continuous for the regression fit output to a categorical output SB = strong binder (< 50 nM) WB = weak binder (> 50 nM and <500 nM) and non-binder (> 500 nM). The r^2 ^is indicated is the metric for how well the particular predictor predicts the values in the training set.

### Neural Network Regression using PCAA

As discussed by Bishop [24] a NN regression of data with continuous responses is effectively a non-linear PLS. In fact, the predecessors of the JMP^® ^platform we used, the statistical analysis programs of SAS http://www.SAS.com have had extensive neural network capabilities for several decades and treat neural networks as simply a special type of regression analysis. Bishop also discusses the use of principal components as a useful, if not essential, adjunct to building appropriately weighted reliable neural networks.

**Figure 3:**
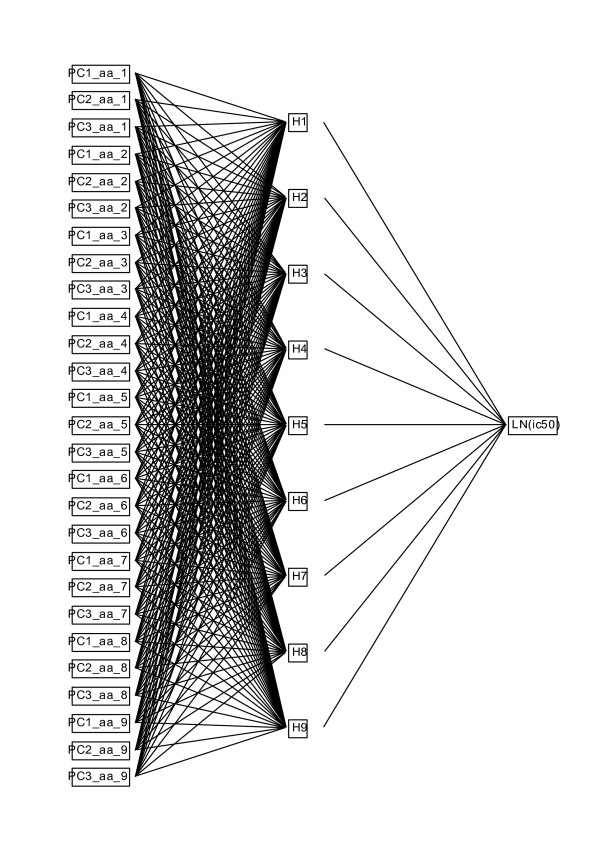
**Layout of the multilayer perceptron neural net used for prediction of MHC binding**. The perceptron has a single input layer of the amino acid principal components, a hidden layer with a number of nodes equal to the binding domain, and a single output layer the natural logarithm of the ic50.

Thus, we submitted the MHC ln(ic50) binding data used in PLS above to the NN platform within JMP^® ^using the z-scale vectors to predict the ln(ic50)) in a simple three-layer perceptron (Figure 3). The non-linear equation that was used by the NN platform for fitting was the logistic function 1/(1+e^x^) that is a well behaved smooth function that asymptotically approaches both a minimum and maximum and at the limit is practically a straight line. One of the key decisions in the design of the NN is to determine the appropriate number of hidden nodes. We chose to use a number of hidden nodes equal to the number of residues in the peptide that interacts with the MHC binding domain (9 hidden nodes for MHC-I and 15 hidden nodes for MHC-II). The rationale was that this symmetry would facilitate comparison of the fit obtained by this method with that described above for the linear PLS fit. It also provides symmetry between the statistical model and the underlying physicochemical model. It should be noted that this number of hidden nodes is actually a rather small number compared to that used by Nielsen and Lund [20] for their substitution matrix NN NetMHCII, used to benchmark our predictors. The JMP^® ^platform has a variety of mechanisms and statistical output for "training" of the NN, in order to control the underlying non-linear regression convergence, to assess the statistical reliability of the output, and to monitor and control overfitting through the use of an overfitting penalty coefficient. We systematically experimented with these control elements to evaluate the quality of the predictions through several cross validation strategies. We found that the presence of peptide subsets with different numbers of peptides, some having radically different mean affinities in the predictors (detected as latent factors in the PLS), are also somewhat problematic for random selection of training subsets during cross validation. The results of two different strategies are reported here. The two different models are referred to as Method 1 and Method 2.

In Method 1 multiple "tours" (different random seeds) of a random holdback strategy were used. Examination of the residuals in the various hyperplanes was used to examine the residuals of these fits. In as much as the three principal components we used for the model account for approximately 90% of variance in the underlying physical properties, we set the overfitting penalties to target an r^2 ^of 0.9. For benchmarking, the prediction models the IEDB datasets downloaded from CBS were contemporaneously submitted to the webservers for NetMHCII (version 2.0) and NetMHCIIPan (version 1.0) at CBS [25-28]. The performance of Method 1 is compared to the PLS model and the output of the servers at CBS in Table 2. As described above for the PLS, both an r^2 ^comparing the fit and a categorical transformation were used to make the comparisons.

The predictions produced by Method 1 and its ability to generalize in the training sets compared favorably to NetMHCII (Table 2) evaluated either as a continuous fit or as a categorical classifier. The statistical metrics associated with the model suggested that some overfitting was likely occuring with this model and therefore a second method (Method 2) was developed.

In Method 2 the prediction models were produced through the use multiple random subsets of the training set each producing a unique set of prediction equations. For example, nine random selections of 2/3 of the training set produces nine sets of prediction equations where each of the peptides will have been used six times in combinations with different peptide cohorts. The predictions of these equations were averaged to produce a mean estimate as well as a standard error of the mean. The coefficient of variation gives an estimate of the variation in the estimates. Results with two differently sized randomly selected subsets of the IEDB training sets are shown in Table 3.

**Table 3:** Coefficient of variation of the mean estimate of the LN(ic50) for different alleles of human MHC-II using two different schemes for cross validation.

Allele	Training	Random 1000	Training
	
	9 × 67% (1)	9 × 67% (2)	9 × 50% (3)
**DRB1_0101**	10.4%	14.4%	17.8%
**DRB1_0301**	6.2%	6.2%	7.4%
**DRB1_0401**	9.5%	9.5%	6.6%
**DRB1_0404**	7.3%	22.0%	9.4%
**DRB1_0405**	7.9%	7.3%	9.3%
**DRB1_0701**	4.8%	10.0%	12.4%
**DRB1_0802**	7.6%	7.0%	8.5%
**DRB1_0901**	12.6%	9.4%	12.9%
**DRB1_1101**	8.3%	7.6%	10.2%
**DRB1_1302**	6.7%	6.6%	8.5%
**DRB1_1501**	10.5%	8.3%	10.4%
**DRB3_0101**	4.4%	4.5%	5.4%
**DRB4_0101**	8.6%	6.9%	9.8%
**DRB5_0101**	12.5%	8.9%	13.8%
	
**Average**	**8.4%**	**9.2%**	**10.2%**

The training dataset used was the IEDB dataset (Additional File 4; Table S3b). The random dataset consisted of 1000 15-mers drawn from the surfome and secretome of the proteome of *Staphylococcus aureus *COL Genbank NC_002951. (1) A random 2/3 of the data set was selected 9 times to produce 9 sets of prediction equations. Each peptide in the set was used 6 times in combination with other peptides in the training set. (2) Equations from (1) were used to predict the LN(ic50) of the random peptides. (3) As in (1) but half of the training set was used to develop the equations.

**Figure 4:**
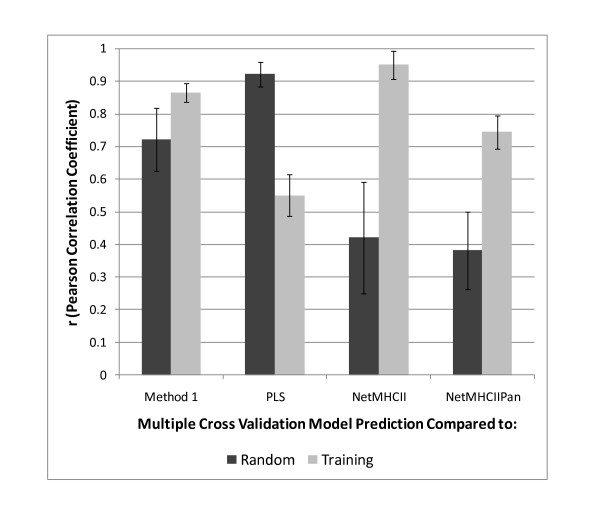
**Comparisons of different prediction schemes for prediction of MHC-II binding affinity**. Comparison of the perfomance of 3 different NN predictors and PLS with the IEDB training set and a random set of 15-mer peptides drawn from the proteome of *Staphylococcus aureus *COL. The mean estimate of the NN described as Method 2 in the text is used as the base comparator. Comparisons are based on the Pearson correlation coefficient (r) of the predicted ln(ic50) as a metric. The error bar is the standard deviation of the r obtained for the 14 different MHC-II alleles. See Additional File 7; Table S5 for detail.

Having five prediction methods based on different underlying predictors, substitution matrices for NetMHCII and NetMHCIIPan and physical properties of amino acids for PLS, Method 1 and Method 2 described above provided an opportunity to examine the comparative performance of the different prediction methods with both the IEDB training sets as well as with other peptides. This was done by creating a test set of 1000 15-mer peptides selected at random from the proteome of *Staphylococcus aureus *COL (Genbank NC_002951). This random test set was submitted to each of prediction tools and the results tabulated for comparison. Figure 4 shows the results of comparisons of the different methods with Method 2 as the base method, using the Pearson correlation coefficient of the predictions as the metric for comparison for the training sets (see additional file 7 for detail). Method 1, NetMHCII and NetMHCIIPan all produce highly correlated predictions, the highest correlations being between Method 2 and NetMHCII. The results of evaluation using categorical predictors gave comparable results (not shown).

As with the training set, the correlated response of between Method 2 and Method 1 is also seen for the random peptide set. Table 3 also shows the comparison of Method 2 with both the training set and the random set. Interestingly, with the random set the correlation with PLS is substantially better than for the training set, however the correlation between Method 2 and both NetMHCII and NetMHCIIPan is diminished. Also, the correlation coefficients of the later two prediction methods show a higher degree of variability.

### MHC-I predictions

Similar comparisons to those described above for MHC-II were conducted with the MHC-I datasets (results not shown). Distributional issues are very common in the MHC-I datasets, like those shown in Figure 2A., in which a clustering of identical ic50 values occur probably as a result of experimental limitations. For the MHC-I, final prediction r^2 ^values are somewhat lower, in the range of 0.7 - 0.85. Visualization of the VIP from the PLS analysis is shown below. As seen with the MHC-II alleles, the PLS analysis suggested the presence of several latent factors in the datasets.

### Visualization of the Variable Importance Projection

**Figure 5:**
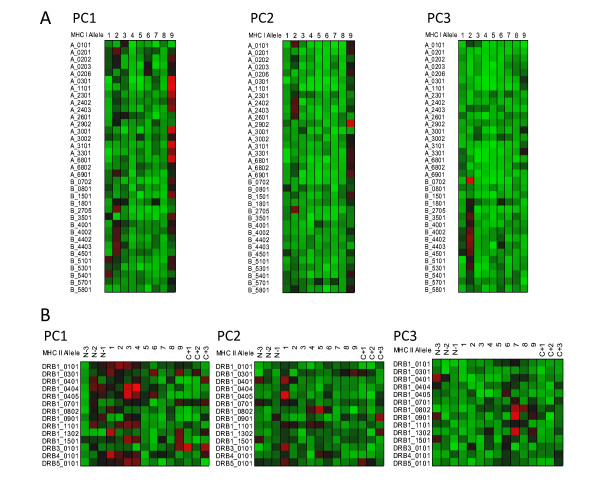
**Visualization of peptide binding to MHC-I and MHC-II**. Variable importance projection (VIP) of the PLS regression prediction of ln(ic50) of peptide binding by using the first three principal components of the amino acids in each of the amino acids in the 9-mer as predictors for MHC-I (5A)and 15-mer for MHC-II (5B). Coloration is uniform over all cells in the matrix for each principal component (a copy of this figure with details of color scaling can be found in Additional File 8; Figure S5). The colors compare the relative importance of the particular numbered residue of the binding domain among all of the MHC alleles indicated. (PC1) Principal component 1 (polarity correlate), (PC2) Principal component 2 (size correlate), and (PC3) Principal component 3 (elctronic correlate). Cells in the matrix with VIP >1 are the most relevant in explaining the binding affinity. The particular MHC allele in each row is indicated on the left. A 9 amino acid binding domain is shown using the standard for the MHC binding groove numbered N-terminus to C-terminus 1 through 9 for MHC-I. A 15 amino acid binding domain is shown using the standard for the MHC-II binding groove numbered N-terminus to C-terminus 1 through 9 flanked by 3 amino acids on the N-terminus and C-terminus.

**Figure 6:**
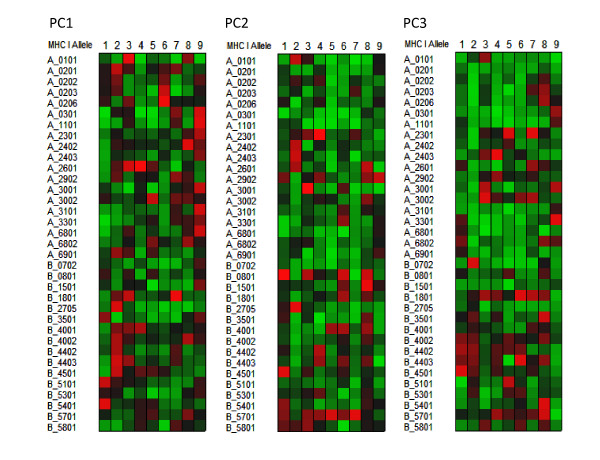
**Visualization of the contribution of the different physical properties of amino acid to the peptide binding to MHC-I**. Variable importance projection (VIP) of the PLS regression prediction of ln(ic50) of peptide binding by using the first three principal components of the amino acids in each of the amino acids in the 9-mer as predictors (PC1) Principal component 1, polarity correlate; (PC2) principal compent 2, size correlate, (PC3) principal component 3, electronic correlate. The colors compare the relative importance of the particular numbered residue of the binding domain among the MHC-I alleles indicated. Cells in the matrix with VIP >1 are the most relevant in explaining the binding affinity. Coloration is column-relative for each position in the binding domain (a copy of this figure with details of color scaling can be found in Additional File 8; Figure S6). The particular MHC-I allele in each row is indicated on the left. A 9 amino acid binding domain is shown using the standard for the MHC binding groove numbered N-terminus to C-terminus 1 through 9.

**Figure 7:**
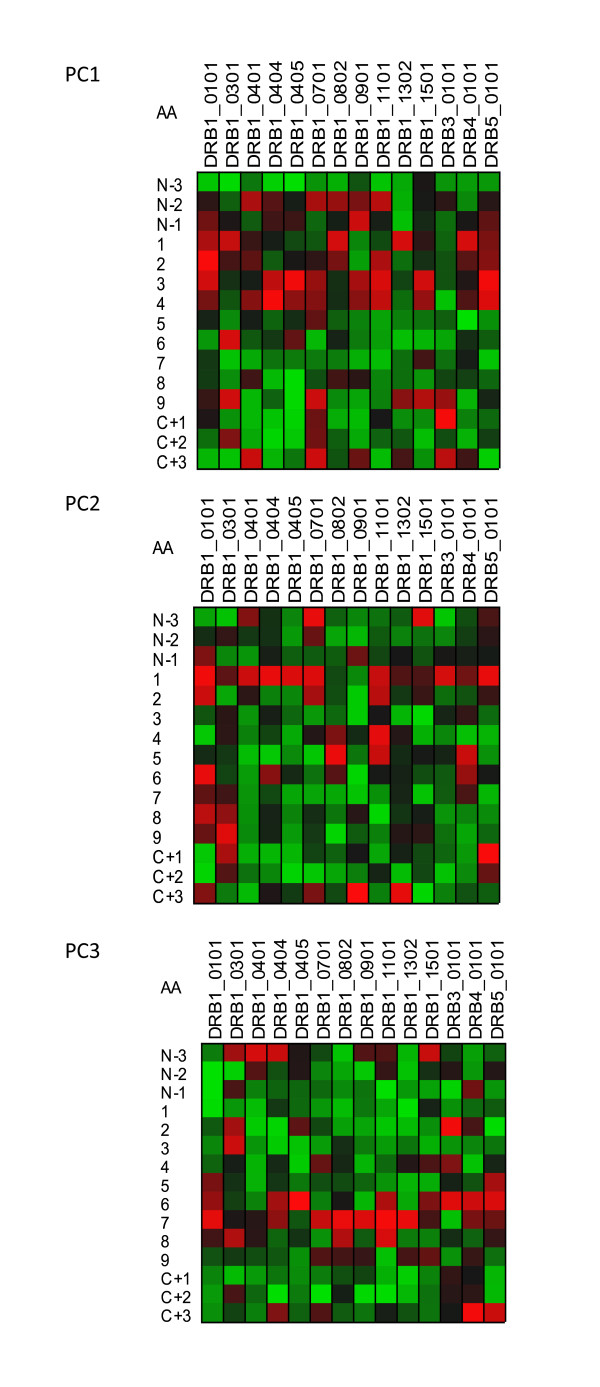
**Visualization of the contribution of the different physical properties of amino acid to the peptide binding to MHC-II**. Variable importance projection (VIP) of the PLS regression prediction of ln(ic50) of peptide binding using the first three principal components of each of the amino acids in the 15-mer as predictors. (PC1) Principal component 1, polarity correlate; (PC2) principal compent 2, size correlate, (PC3) principal component 3, electronic correlate. Coloration is column-relative indicated by the scales for each position in the binding domain (a copy of this figure with details of color scaling can be found in Additional File 8; Figure S7). The amino acid in the binding domain of the particular MHC-II allele in each column is indicated on the left. A 15-amino acid binding domain is shown using the standard for the MHC binding groove numbered N-terminus to C-terminus 1 through 9 along with the additional 3 N-terminal and 3 C-terminal residues. The colors compare the relative importance of the particular numbered residue of the binding domain among the MHC-II alleles indicated. Cells in the matrix with VIP >1 are the most relevant in explaining the binding affinity.

**Figure 8:**
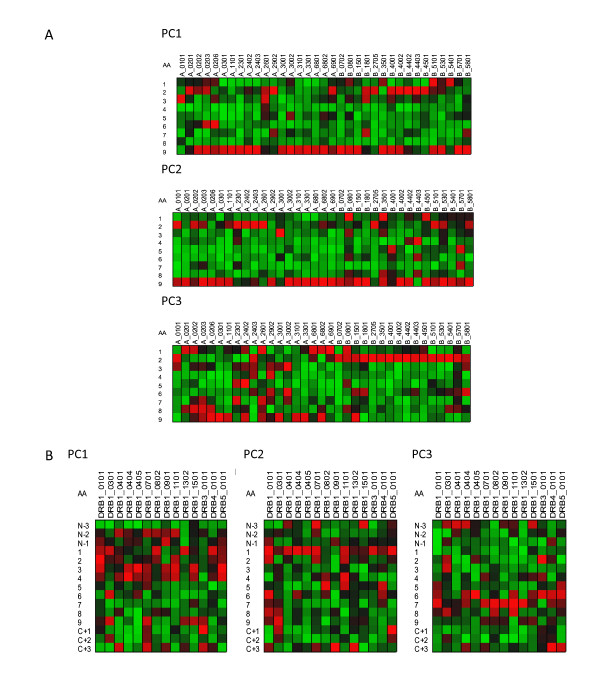
**Visualization of the contribution of different residues in MHC binding**. Variable importance projection (VIP) of the PLS regression prediction of ln(ic50) of peptide binding using the first three principal components of each of the amino acids in the 9-mer as predictors (PC1) Principal component 1, polarity correlate; (PC2) principal compent 2, size correlate, (PC3) principal component 3, electronic correlate. Coloration is column-relative indicated by the scales for each position in the binding (a copy of this figure with details of color scaling can be found in Additional File 8; Figure S8). The colors compare the relative importance of the particular numbered residue of the binding domain among the MHC-I alleles indicated. Cells in the matrix with VIP >1 are the most relevant in explaining the binding affinity. The amino acid in the binding domain of the particular MHC-I allele (A) or MHC-II (B) in each column is indicated on the left. A 9 amino acid binding domain is shown using the standard for the MHC binding groove numbered N-terminus to C-terminus 1 through 9. For MHC-II three additional amino acids are added to each the N-terminus and C-Terminus.

Computation of the VIP provides a means of estimating the relative importance of predictors to the overall model and as such is a very useful aspect of a PLS analysis. In the case of the peptide datasets used here, where the predictors are conceptual physicochemical proxies, the VIP provides a statistical view of the underlying physical interactions between the peptide and the MHC binding domains. As a visualization paradigm for the VIP we use a "heat plot", commonly used in other areas of systems biology. In these relatively simple plots, each cell represent tens of thousands of statistical comparisons reduced to a single metric and displayed in a uniformly scaled graphic system that facilitates visual comprehension [29]. All of the plots in Figures 5, 6, 7, and 8 are produced from two VIP matrices (the matrices can be found in the Additional File 3; Tables S3a and Additional File 4; Table S3b), one for MHC-I binding and the other for MHC-II, that have been colorized in different ways to accentuate different potential physicochemical properties and to summarize all binding reactions of all peptides in the datasets used to derive the predictions described above. Any value in the VIP matrices >1 are interpreted as being the most relevant in explaining the binding affinity. In Figures 5, 6, 7, and 8 the matrices are colored by three different methods which provide different views of the underlying physicochemical properties and interactions between the peptide and binding site. Each of the underlying physical properties are on individual plots; Additional File 8; Figures S5, S6, S7, and S8 provide copies of Figures 5, 6, 7, and 8 that include the "thermometers" for the heat plots that detail how the numerical information in the VIP matrix was color scaled. We designate the MHC binding pocket to consist of 9 zones of interactions given the label P1 to P9 based on MHC-I. Peripheral to this, the MHC-II molecules comprises 3 additional residues on each side (N-3, N-2, N-1 and C+1, C+2 and C+3) to make up the 15-mer. In MHC-II molecules the ends of the binding pocket (groove) are open and longer peptides can interact with additional residues.

Figure 5 shows the VIP of the PLS regression prediction of ln(ic50) of peptide binding by using the first three principal components of the amino acids in each of the amino acids in the 9-mer as predictors for MHC-I (5a) and 15-mer for MHC-II (5b). In these Figures the coloration of the plot is uniform over all cells in the matrix (complete scaling information is found in the Additional File 8; Figure S5). The colors compare the relative importance of the particular numbered residue of the binding domain among all of the MHC alleles indicated. As the selection of peptide sequences were highly non-random it is likely that the coloration seen is a combination of two factors, the prior knowledge of the experimentalists use in designing the amino acid combinatorials in the peptides and the actual physicochemical properties. The coloration is consistent with the amino acid composition thought to provide the best fit into the binding groove. The VIP provides an insight into the potential physicochemical interactions in the binding groove of MHC molecules which have not been studied as intensely. The relative importance of the polarity of position 9 for the MHC-I locus A binding is seen. PC3, the third ranked principal component electronic physical correlate, emerges as important in position 2 of MHC-I locus B alleles. In Figures 6 and 7 the coloration emphasizes a different aspect of the binding interaction of MHC-I (Figure 6) and MHC-II (Figure 7). In these two Figures the color scaling compares the relative importance of the particular numbered residue of the binding domain among the all of the alleles indicated. Again, cells in the VIP matrix with a value >1 are the most relevant in explaining the binding affinity. Coloration is column-relative for each position in the binding domain. One position of note in Figure 7 is the strong signal of PC1 (the polarity correlate) for C+1 position for DRB3*0101. Giving credence to the properties visualized, a recent paper of Parry [30] using an entirely different methodology, suggested a major role of P10 (the binding zone located one amino acid position toward the C-terminus and equivalent to C+1 in our notation) in the binding of peptides to DRB3*0101. For different MHC-II alleles the possible role of each of the three physical properties in the overall binding reaction of those N-terminal and C-terminal residues outside the core binding pocket is suggested by these Figures.

Figure 8 provides a visualization only for MHC-I; output for MHC-II is included only in the Additional File 8; Figure S8. Coloration scaling is column-relative for each position in the binding domain. The comparison is within MHC-I allele and is repeated for each of the 3 principal components. The colors compare the relative importance of the physicochemical property of each particular numbered residue of the binding domain within each of the MHC-I alleles indicated.

**Figure 9:**
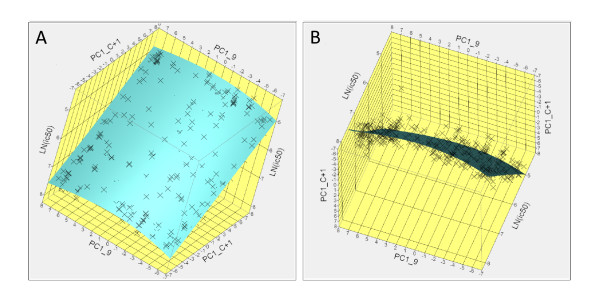
Example of visualization of physicochemical interactions between the peptide and the binding pocket of DRB3*0101. Potential effects of physicochemical interactions on the binding affinity can be explored interactively for all combinations of peptide amino acid and binding groove domain. (A) Inter-relationship of principal property 1 (hydrophobicity) for positions P9 and P(C+1). (B) rotation of the hyperplane of (A) to show the scatter of the residuals of the fit about this hyperplane.

Using the JMP^® ^"contour profiler" it is possible to segregate and visualize of the interaction between any two of the input variables and the output prediction. An example is shown in Figure 9, however a two-dimensional representation does not adequately illustrate the fully interactive demonstration of the physicochemical interactions involved in the binding reactions. Figure 9 is a plot showing a hyperplane of the first principal component predictions for amino acid P9 and C+1 (also called P10 by Parry [30]). It shows not only how the polarity of these two residues might play a role in binding, but also how the experimentalist might systematically vary the amino acids in these positions and what would be the expected impact on binding affinity. In the perspective depicted in Panel A the non-randomness of the amino acids used in the training sets is readily apparent. Panel B provides a means of examining of the relative scatter of the actual datapoints around the fitted hyperplane in multidimensional space.

## Conclusions

We have shown the utility of using QSAR concepts that are the backbone of cheminformatics to analyze and build a tool for prediction of MHC binding. Three different prediction schemes are described that use the first three principal components of the physical properties of amino acids as predictors. The first scheme uses a linear PLS approach that is commonly used in QSAR. In addition, two different cross-validation approaches are used to develop NN prediction equations in the JMP^® ^application. Overall, these methods benchmark well against the most recently available substitution matrix predictors when considered as either as categorical predictors or as regression predictors using the IEDB training sets. The three prediction schemes described in this paper also perform consistently with both the training sets and a random comparator test set of 15-mer peptides. Unlike their performance with the training sets, NetMHCII and NetMHCIIPan do not perform as consistently with the random test set. Given the relatively good agreement between the methods for the IEDB training sets this result with the random set is unanticipated and the reason for the disparity is unknown. We suspect one possibility might be the implicit non-randomness of the peptide structures synthesized to create the training sets themselves. As we noted, within the IEDB datasets there are clusters of peptide subsets with unique and statistically separable characterisitics. The peptide structures designed for reliable predictions of binding with relatively small sets of peptides might be so unique as to provide relatively poor training for random peptides such as those that might be encountered from a proteome. It is also conceivable that this is but a different facet of the issues noted by El-Manzalawy *et al *[14] for these datasets.

Symmetry between the statistical models and the underlying physicochemical interactions provides a unique way to use a large number of binding measurements to gain insight into the binding reactions and results in a statistical docking akin to molecular docking done *in silico *structural studies which are based on physical properties at the atomic level [31]. These statistical models complement and corroborate inferences drawn from other physical measurements of the interactions between the peptide and the MHC binding domain. Unlike matrix approaches which view the binding site as a static entity without interactions between adjacent amino acids (see Parry [30] for discussion) the approach adopted in this paper makes no assumptions about the interaction: a peptide of a fixed length having three major physicochemical properties, interacts with an MHC binding domain, and binds to it with a certain affinity. While no "anchor" residue is predicted, it is conceivable that the VIP might be implicitly providing an equivalent of an anchor residue score as can be seen by comparing the scales of the heat plot thermometers in the Additional File 8; Figures S5-S8.

By use of all the binding data in a PLS model, the VIP suggest that the first two residues outside the MHC-II binding pocket at the N terminus and C terminus play significant roles in the binding interaction. This finding corroborates and extends the work of Parry [30].

The true power of PLS and QSAR approaches have been in their applications to molecular and experimental design. In this regard the concepts described herein might provide a context within which to design molecular dynamic simulations of the physicochemical interplay between a peptide and the MHC binding site domains.

An important feature of our approach is that it makes it possible to analyze entire proteomes comprising over a million binding peptides in real-time on a desktop (or laptop) computer. Coupled with the data visualization capabilities [8], it enables new insights into the underlying physicochemical characteristics of peptide binding to the MHC molecules and should assist in future experimental design. In the companion paper we describe the use of these tools in combination with other predictors to create an integrated epitope prediction platform capable of proteome-scale analyses.

## Methods

### Data sources

Amino acid physical properties were retrieved from the repository at the Swiss Institute of Bioinformatics: http://expasy.org/tools/protscale.html

MHC-I and MHC-II peptide binding datasets were retrieved from the repository at Center for Biological Sequence Analysis http://www.cbs.dtu.dk and IEDB http://www.IEDB.org.

A random dataset of 1000 15-mer peptides was selected from the surfome and secretome of *Staphylococcus aureus *COL (Genbank ID NC_002951) (Additional File 9; Table S6).

### Internet data analysis and manipulation

Web servers for benchmark comparisons NetMHCII version 2.1, NetMHCIIPan 1.0 http://www.cbs.dtu.dk/services/NetMHCII/. Some testing was done with the MHCserver but there were no extensive or systematic comparisons to NetMHC 3.0.

### Workstation applications

The Partial Least Squares and Neural Network platforms in JMP^® ^8.02 and JMP^® ^Genomics 4.2 were used for all local calculations and data manipulation (SAS Institute Inc. Cary, NC).

The statistical background for the JMP^® ^platforms is found in: JMP^® ^8 Statistics and Graphics Guide, SAS Institute Inc., Cary, NC, USA (ISBN 978-1-59994-923-9).

### Variable influence

For a given PLS dimension **a**, (VIN)_ak_^2 ^is equal to the squared PLS weight (w_ak_)^2 ^of that term, multiplied by the explained sums of squares (SS) of that PLS dimension [2,3]. The accumulated (overall PLS dimensions) value,

$$VIP_{ak}=\sum_{a}{\left(VIN\right)}_{k}$$

is then divided by the total explained SS by the PLS model and multiplied by the number of terms in the model. The final VIP is the square root of that number. The formula can also be expressed as follows:

$$VIP_{ak}=\sqrt{\left\{\sum_{a=1}^{A}\left(w_{ak}^{2}*\left(SSY_{a-1}-SSY_{a}\right)\right)*\frac{K}{\left(SSY_{0}-SSY_{A}\right)}\right\}}$$

The SS of all VIP is equal to the number of terms in the model and hence the average VIP is equal to 1. The VIP of one term can be compared to others and terms with the larger VIP, larger than 1, are the most relevant for explaining the response. The VIP were computed within the PLS platform of JMP^®^.

## Abbreviations

AROC: area under the receiver operator characteristic curve; CBS: Center for Biological Sequence Analysis; IEDB: Immune Epitope Database; ic50: inhibitory concentration 50%; ln(ic50): natural logarithm of the ic50; NN: Neural Net; PCAA: Principal Component Amino-acid Analysis; PLS: Partial Least Squares; PSSM: Position sensitive substitution matrices; QSAR: Quantitative Structure Activity Relationships; VIP: Variable importance projection.

## Competing interests

Drs. Bremel and Homan are founding scientists and employees of ioGenetics LLC. Patent applications have been filed on components of the technology described herein.

## Authors' contributions

RDB designed and performed computational analysis. Both authors conceived the study design, contributed to its execution, and drafted the manuscript. Both authors read and approved the final manuscript.

## Supplementary Material

Additional file 1 **Table S1: Listing of internet sites with relevant computing and resource sites**. Click here for file

Additional file 2 **Table S2: Physicochemical Properties of amino acids used for computing the principal components**. Click here for file

Additional file 3 **Table S3a: Peptides used for Neural Network training: MHC-I**. Click here for file

Additional file 4 **Table S3b: Peptides used for Neural Network training: MHC-II**. Click here for file

Additional file 5 **Table S4a: VIP MHC-I**. Click here for file

Additional file 6 **Table S4b: VIP MHC-II**. Click here for file

Additional file 7 **Table S5: Correlations among different prediction schemes using the IEDB dataset**. Click here for file

Additional file 8 **Figures S5, S6, S7, and S8: Correspond to Figures **5, 6, 7, **and **8**and contain additional detail**. Click here for file

Additional file 9 **Table S6: Random 15-mers from *Staphylococcus aureus *COL along with the output from multiple prediction schemes**. Click here for file
