# Supplementary material for: An integrated approach to epitope analysis I: Dimensional reduction, visualization and prediction of MHC binding using amino acid principal components and regression approaches
Source: Immunome Res. 2010 Nov 2;6:7. doi: 10.1186/1745-7580-6-7 (PMC2990731; doi:10.1186/1745-7580-6-7)
Supplement: Additional file 1 — Table S1: Listing of internet sites with relevant computing and resource sites. [file 1745-7580-6-7-S1.PDF]

Additional Table S1. Listing of internet sites with relevant computing and resource sites

General immunology resources	http://www.immuneepitope.org/
Amino acid physical properties	http://expasy.org/tools/protscale.html
Training sets	http://www.immuneeepitope.org/links/
Web NN & Training sets	http://www.cbs.dtu.dk/suppl/immunology/NetMHCII-2.0.php
Web NN & training sets	http://www.cbs.dtu.dk/services/NetMHC/
Training Sets	http://bio.dfci.harvard.edu/DFRMLI/
Training Sets	http://www.syfpeithi.de/
Philius protein topology predictor	http://www.yeastrc.org/philius
Phobius protein topology predictor	http://phobius.binf.ku.dk/

See also:

Salimi N, Fleri W, Peters B, Sette A: **Design and utilization of epitope-based databases and predictive tools**. *Immunogenetics* 2010, **62:**185-196.
