# Supplementary material for: An integrated approach to epitope analysis I: Dimensional reduction, visualization and prediction of MHC binding using amino acid principal components and regression approaches
Source: Immunome Res. 2010 Nov 2;6:7. doi: 10.1186/1745-7580-6-7 (PMC2990731; doi:10.1186/1745-7580-6-7)
Supplement: Additional file 2 — Table S2: Physicochemical Properties of amino acids used for computing the principal components. [file 1745-7580-6-7-S2.PDF]

Additional Table S2: Physicochemical Properties of amino acids used for computing the principal components.

1	Polarity.	[1]
2	Polarity (p).	[2]
3	Optimized matching hydrophobicity (OMH).	[3]
4	Hydropathicity.	[4]
5	Hydrophobicity (free energy of transfer to surface in kcal/mole).	[5]
6	Hydrophobicity scale based on free energy of transfer (kcal/mole).	[6]
7	Hydrophobicity (delta G1/2 cal)	[7]
8	Hydrophobicity scale (contact energy derived from 3D data).	[8]
9	Hydrophobicity scale (pi-r).	[9]
10	Molar fraction (%) of 2001 buried residues.	[10]
11	Proportion of residues 95% buried (in 12 proteins).	[11]
12	Free energy of transfer from inside to outside of a globular protein.	[10]
13	Hydration potential (kcal/mole) at 25øC.	[12]
14	Membrane buried helix parameter.	[13]
15	Mean fractional area loss (f) [average area buried/standard state area].	[14]
16	Average area buried on transfer from standard state to folded protein.	[15]
17	Molar fraction (%) of 3220 accessible residues.	[10]
18	Hydrophilicity.	[16]
19	Normalized consensus hydrophobicity scale.	[17]
20	Average surrounding hydrophobicity.	[18]
21	Hydrophobicity of physiological L-alpha amino acids	[19]
22	Hydrophobicity scale (pi-r)2.	[20]
23	Retention coefficient in HFBA.	[21]
24	Retention coefficient in HPLC, pH 2.1.	[22]
25	Hydrophilicity scale derived from HPLC peptide retention times.	[23]
26	Hydrophobicity indices at ph 7.5 determined by HPLC.	[24]
27	Retention coefficient in TFA	[25]
28	Retention coefficient in HPLC, pH 7.4	[22]
29	Hydrophobicity indices at pH 3.4 determined by HPLC	[24]
30	Mobilities of amino acids on chromatography paper (RF)	[26]
31	Hydrophobic constants derived from HPLC peptide retention times	[27]

Reference List

1. Zimmerman JM, Eliezer N, Simha R: **The characterization of amino acid sequences in proteins by statistical methods**. *J Theor Biol* 1968, **21:**170-201.

2. Grantham R: **Amino acid difference formula to help explain protein evolution**. *Science* 1974, **185:**862-864.

3. Sweet RM, Eisenberg D: **Correlation of sequence hydrophobicities measures similarity in three-dimensional protein structure**. *J Mol Biol* 1983, **171:**479-488.

4. Kyte J, Doolittle RF: **A simple method for displaying the hydropathic character of a protein**. *J Mol Biol* 1982, **157:**105-132.

5. Bull HB, Breese K: **Surface tension of amino acid solutions: a hydrophobicity scale of the amino acid residues**. *Arch Biochem Biophys* 1974, **161:**665-670.

6. Guy HR: **Amino acid side-chain partition energies and distribution of residues in soluble proteins**. *Biophys J* 1985, **47:**61-70.

7. Abraham DJ, Leo AJ: **Extension of the fragment method to calculate amino acid zwitterion and side chain partition coefficients**. *Proteins* 1987, **2:**130-152.

8. Miyazawa S, Jernigan RL: **CHECK Hydrphobicity scale**. *Macromolecules* 1985, **18:**534-552.

9. Roseman MA: **Hydrophilicity of polar amino acid side-chains is markedly reduced by flanking peptide bonds**. *J Mol Biol* 1988, **200:**513-522.

10. Janin J: **Surface and inside volumes in globular proteins**. *Nature* 1979, **277:**491-492.

11. Chothia C: **The nature of the accessible and buried surfaces in proteins**. *J Mol Biol* 1976, **105:**1-12.

12. Wolfenden R, Andersson L, Cullis PM, Southgate CC: **Affinities of amino acid side chains for solvent water**. *Biochemistry* 1981, **20:**849-855.

13. Rao MJK, Argos P: **Amino acid scale: membrane buried helix parameter**. *Biochim Biophys Acta* 1986, **869:**197-214.

14. Rose GD, Geselowitz AR, Lesser GJ, Lee RH, Zehfus MH: **Hydrophobicity of amino acid residues in globular proteins**. *Science* 1985, **229:**834-838.

15. Rose GD, Geselowitz AR, Lesser GJ, Lee RH, Zehfus MH: **Hydrophobicity of amino acid residues in globular proteins**. *Science* 1985, **229:**834-838.

16. Hopp TP: **Use of hydrophilicity plotting procedures to identify protein antigenic segments and other interaction sites**. *Methods Enzymol* 1989, **178:**571-585.

17. Eisenberg D, Schwarz E, Komaromy M, Wall R: **Analysis of membrane and surface protein sequences with the hydrophobic moment plot**. *J Mol Biol* 1984, **179:**125-142.

18. Manavalan P, Ponnuswamy PK: **Hydrophobic character of amino acid residues in globular proteins**. *Nature* 1978, **275:**673-674.

19. Black SD, Mould DR: **Development of hydrophobicity parameters to analyze proteins which bear post- or cotranslational modifications**. *Anal Biochem* 1991, **193:**72-82.

20. Fauchere JL, Charton M, Kier LB, Verloop A, Pliska V: **Amino acid side chain parameters for correlation studies in biology and pharmacology**. *Int J Pept Protein Res* 1988, **32:**269-278.

21. Browne CA, Bennett HP, Solomon S: **The isolation of peptides by high-performance liquid chromatography using predicted elution positions**. *Anal Biochem* 1982, **124:**201-208.

22. Meek JL: **Prediction of peptide retention times in high-pressure liquid chromatography on the basis of amino acid composition**. *Proc Natl Acad Sci U S A* 1980, **77:**1632-1636.

23. Parker JM, Guo D, Hodges RS: **New hydrophilicity scale derived from high-performance liquid chromatography peptide retention data: correlation of predicted surface residues with antigenicity and X-ray-derived accessible sites**. *Biochemistry* 1986, **25:**5425-5432.

24. Cowan R, Whittaker RG: **Hydrophobicity indices for amino acid residues as determined by high-performance liquid chromatography**. *Pept Res* 1990, **3:**75-80.

25. Browne CA, Bennett HP, Solomon S: **The isolation of peptides by high-performance liquid chromatography using predicted elution positions**. *Anal Biochem* 1982, **124:**201-208.

26. Akintola A, Aboderin AA: **An empirical hydrophobicity scale for amino acids and some of its applications**. *Int J Biochem* 1971, **2:**537-544.

27. Wilson KJ, Honegger A, Stotzel RP, Hughes GJ: **The behaviour of peptides on reverse-phase supports during high-pressure liquid chromatography**. *Biochem J* 1981, **199:**31-41.
