# Supplementary material for: An integrated approach to epitope analysis I: Dimensional reduction, visualization and prediction of MHC binding using amino acid principal components and regression approaches
Source: Immunome Res. 2010 Nov 2;6:7. doi: 10.1186/1745-7580-6-7 (PMC2990731; doi:10.1186/1745-7580-6-7)
Supplement: Additional file 8 — Figures S5, S6, S7, and S8: Correspond to Figures 5, 6, 7, and 8and contain additional detail. [file 1745-7580-6-7-S8.PDF]

Additional Figure S5a

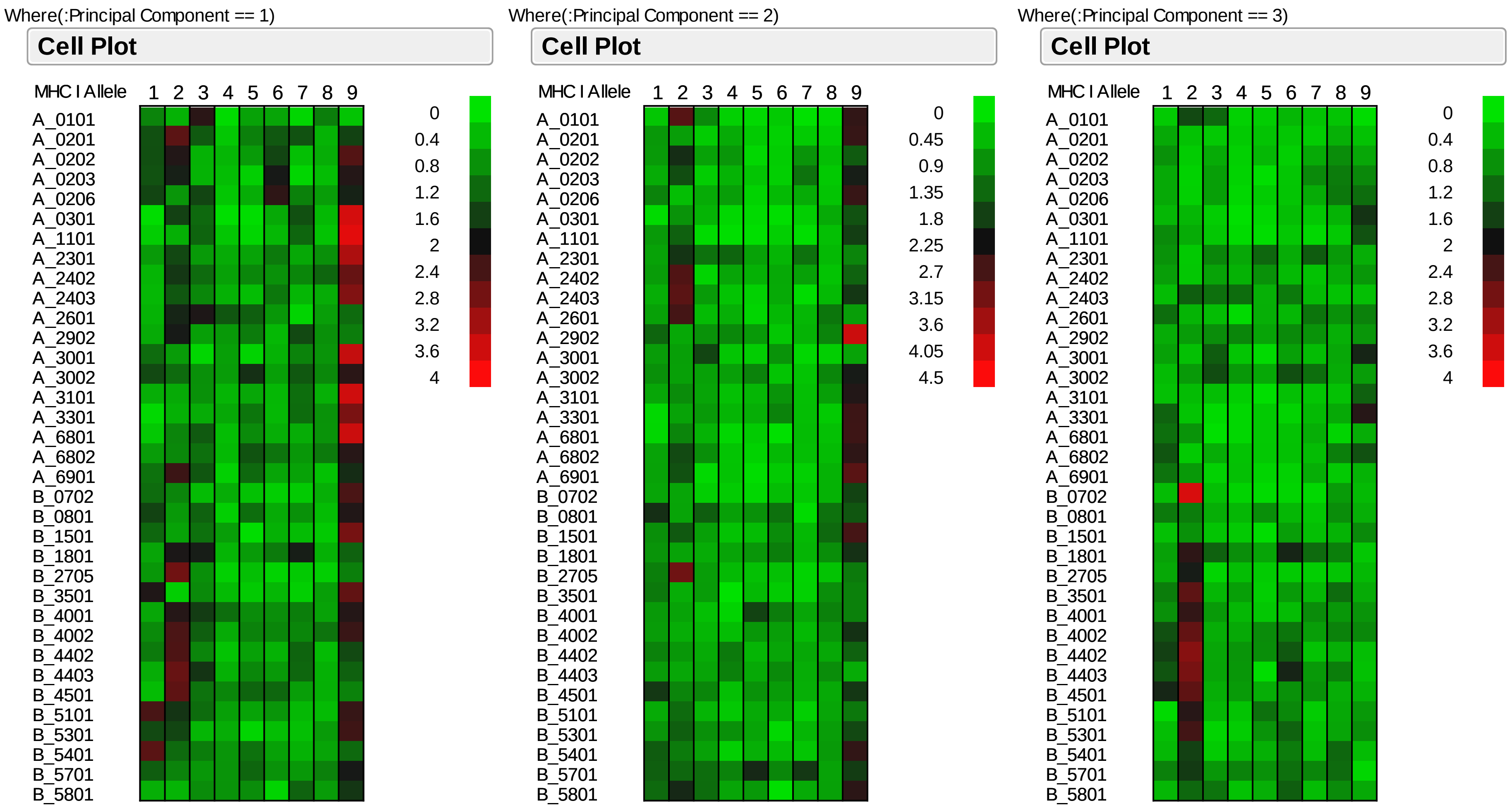

Additional Figure S5b

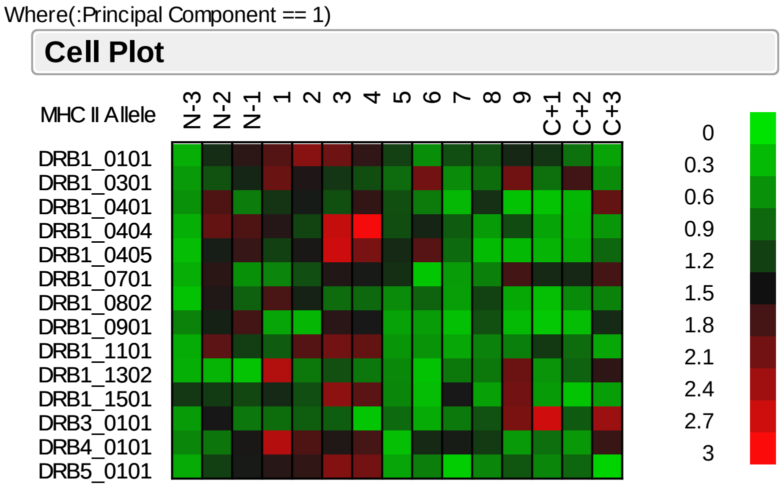

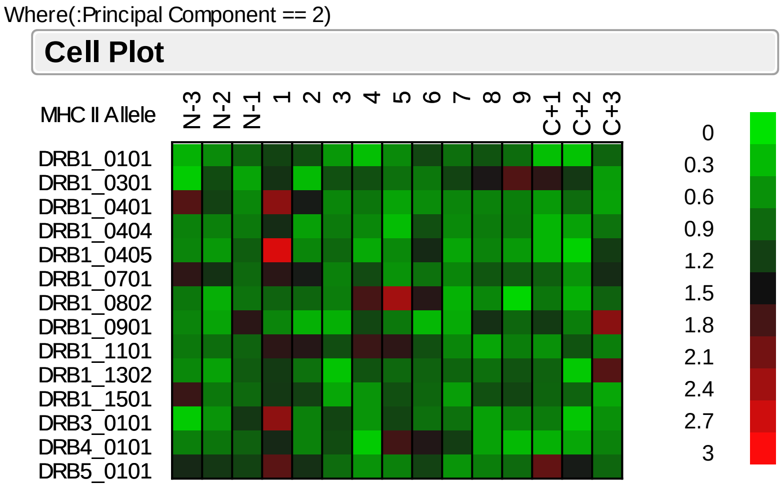

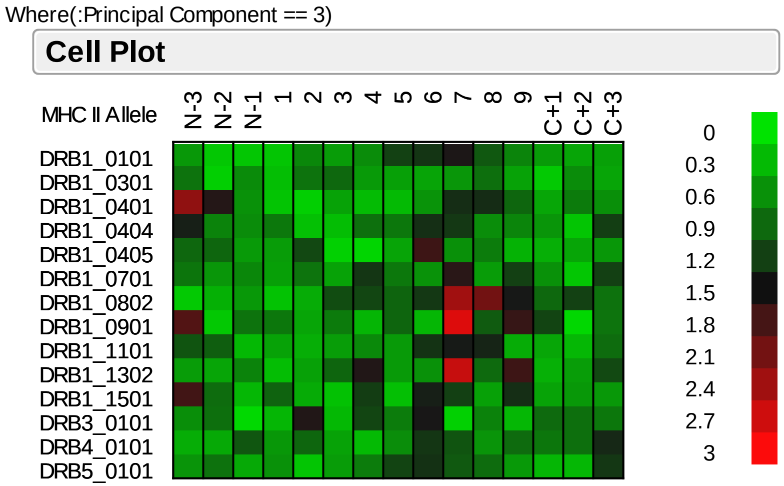

Additional Figure S6_PC1

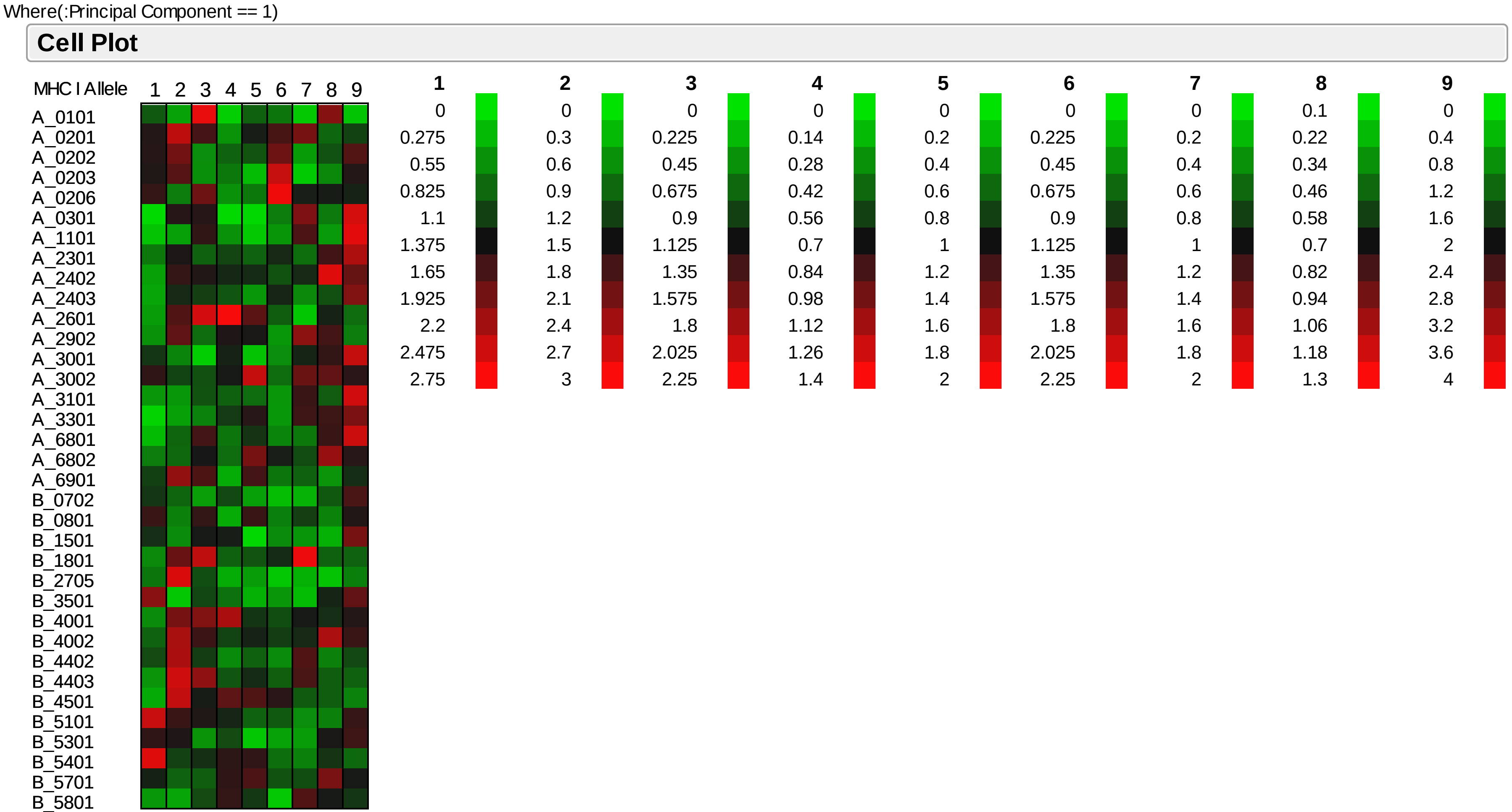

Additional Figure S6_PC2

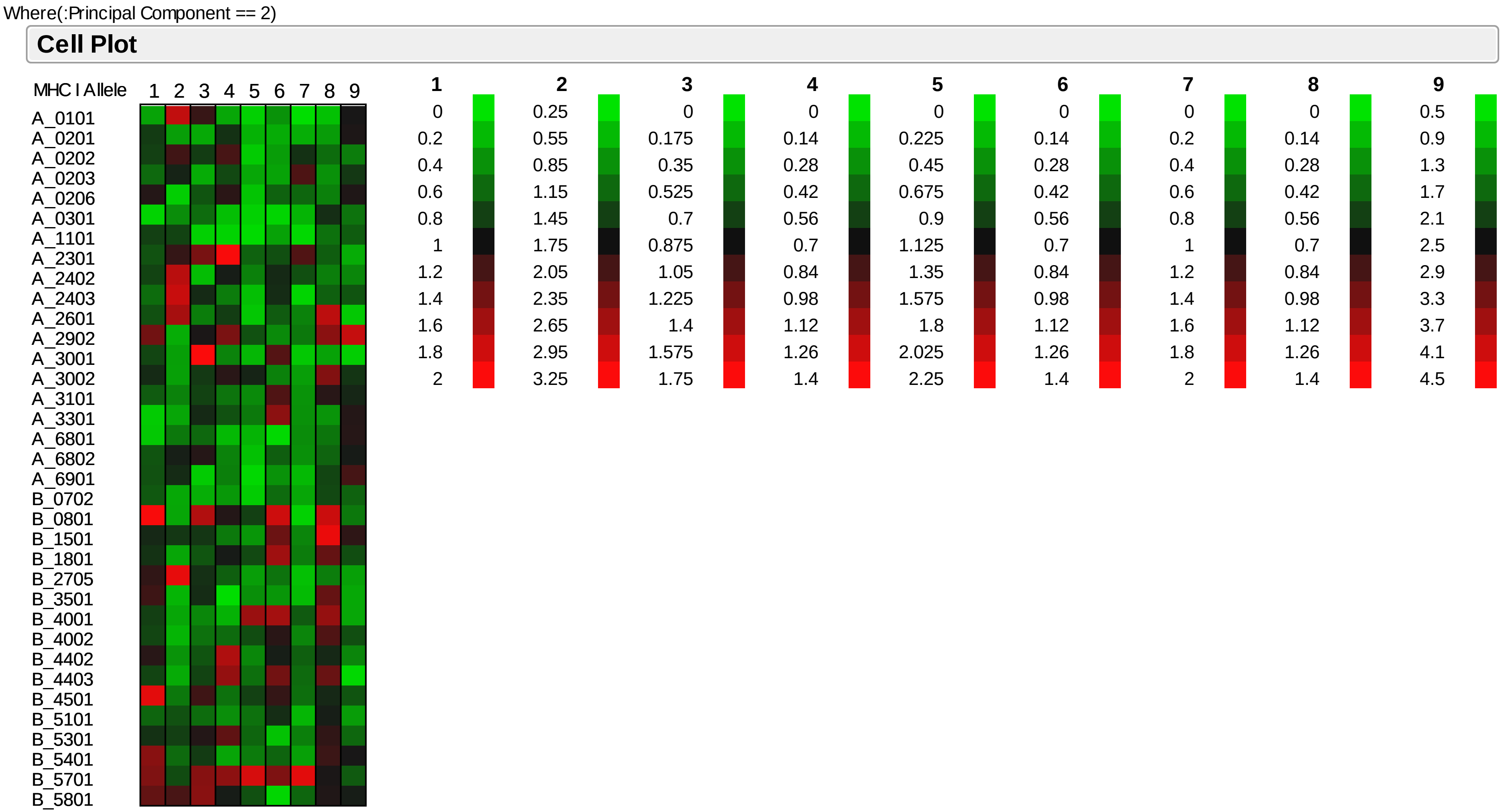

Additional Figure S6_PC3

Where(:Principal Component == 3)

Cell Plot

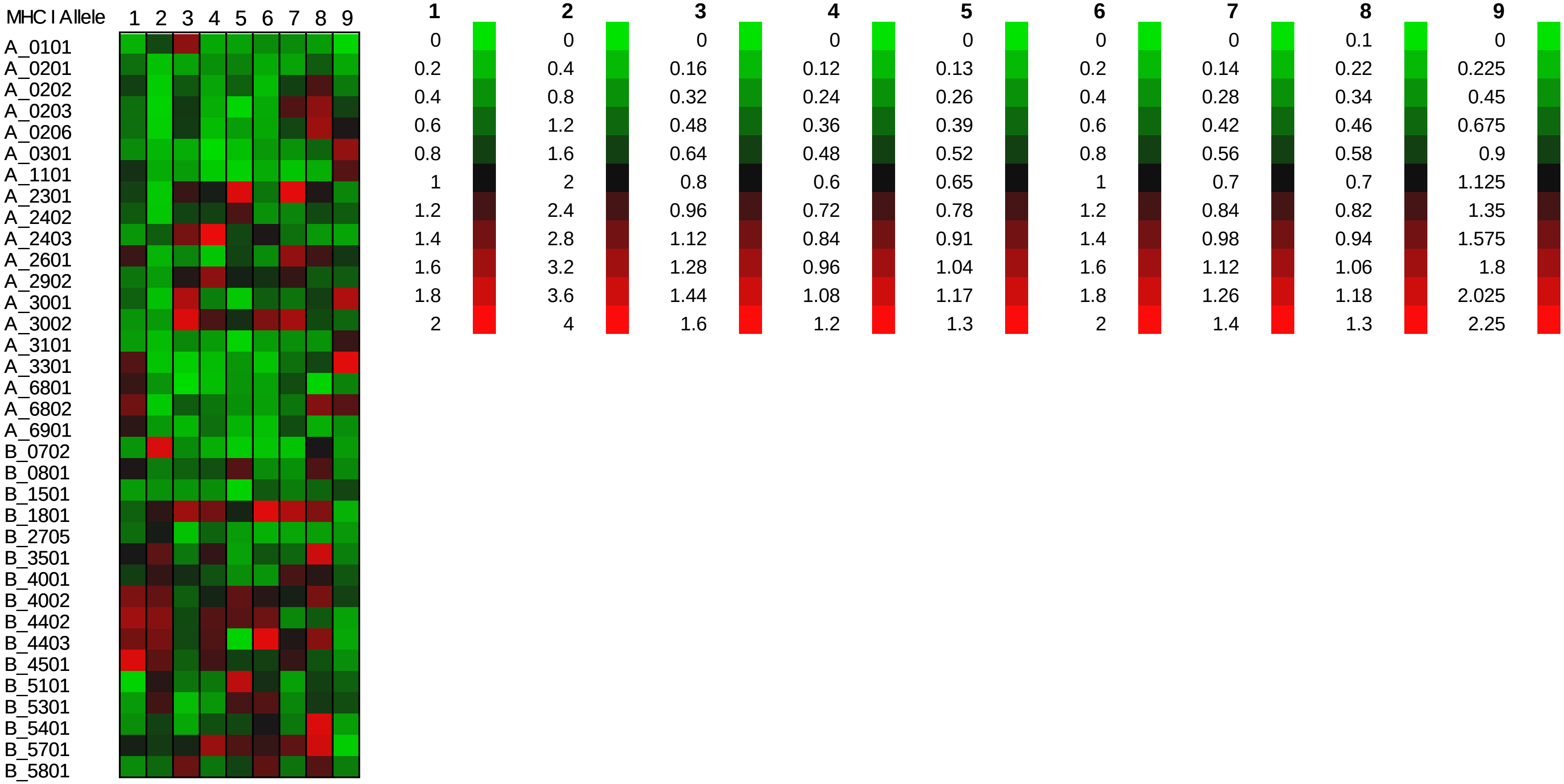

Additional Figure S7

Where(:Principal Component == 1)

Cell Plot

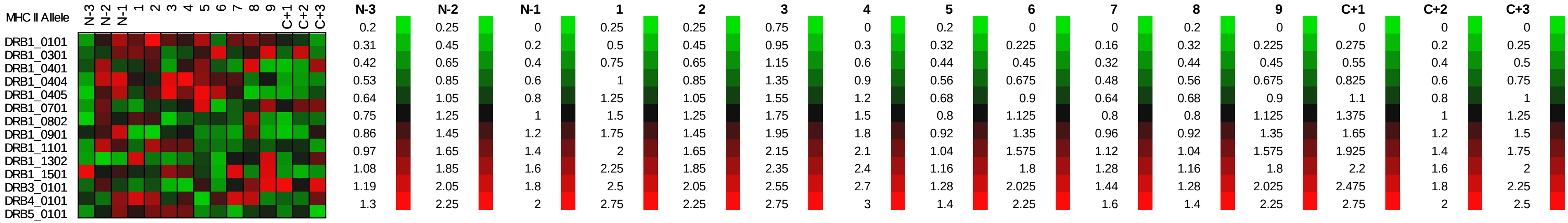

Where(:Principal Component == 2)

Cell Plot

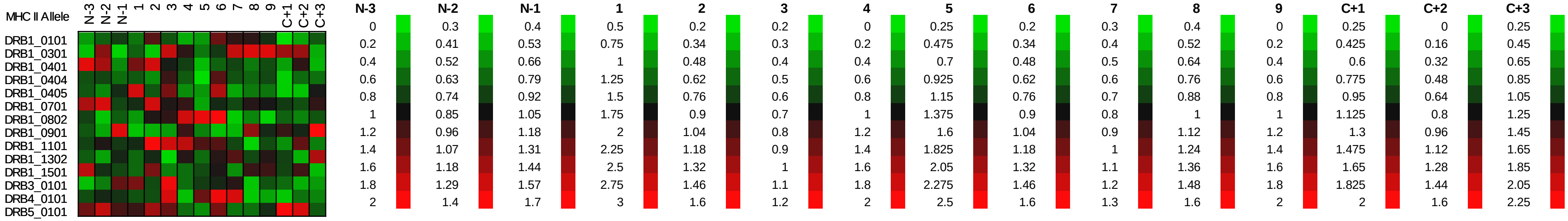

Where(:Principal Component == 3)

Cell Plot

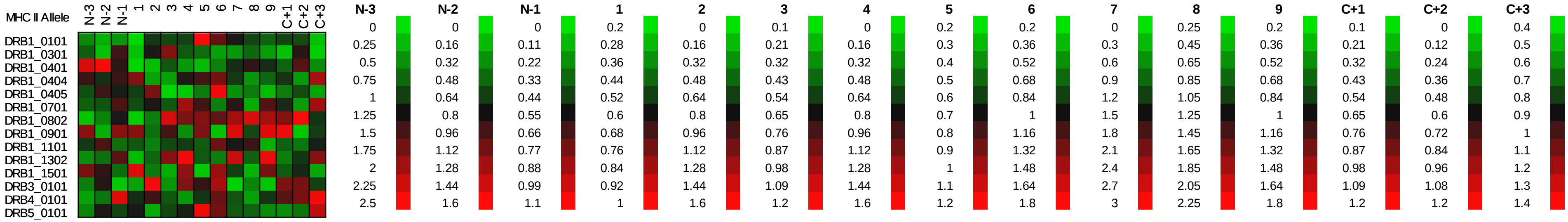

Additional Figure S8_ MHC-I

Where(:Principal Component == 1)

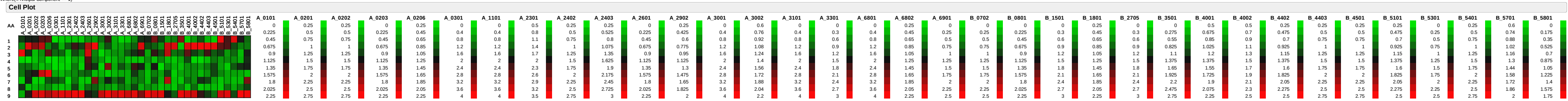

Where(:Principal Component == 2)

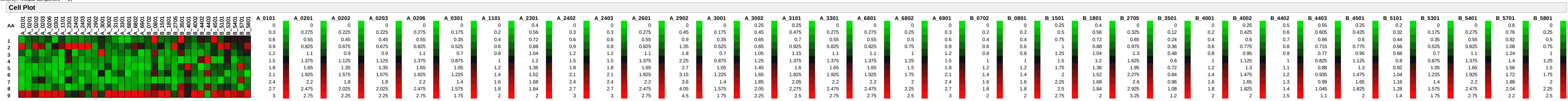

Where(:Principal Component == 3)

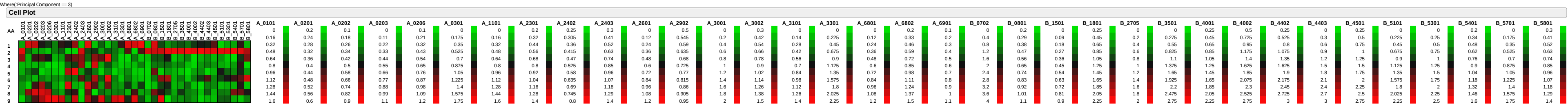

Additional Figure S8_ MHC-II

Where(:Principal Component == 1)

Cell Plot

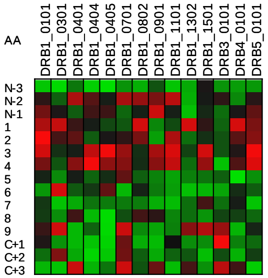

DRB1_0101	DRB1_0301	DRB1_0401	DRB1_0404	DRB1_0405	DRB1_0701	DRB1_0802	DRB1_0901	DRB1_1101	DRB1_1302	DRB1_1501	DRB3_0101	DRB4_0101	DRB5_0101
0.25	0.5	0	0.25	0.25	0	0	0	0.25	0	0	0	0.25	0
0.45	0.675	0.225	0.525	0.5	0.2	0.2	0.2	0.45	0.275	0.25	0.275	0.5	0.225
0.65	0.85	0.45	0.8	0.75	0.4	0.4	0.4	0.65	0.55	0.5	0.55	0.75	0.45
0.85	1.025	0.675	1.075	1	0.6	0.6	0.6	0.85	0.825	0.75	0.825	1	0.675
1.05	1.2	0.9	1.35	1.25	0.8	0.8	0.8	1.05	1.1	1	1.1	1.25	0.9
1.25	1.375	1.125	1.625	1.5	1	1	1	1.25	1.375	1.25	1.375	1.5	1.125
1.45	1.55	1.35	1.9	1.75	1.2	1.2	1.2	1.45	1.65	1.5	1.65	1.75	1.35
1.65	1.725	1.575	2.175	2	1.4	1.4	1.4	1.65	1.925	1.75	1.925	2	1.575
1.85	1.9	1.8	2.45	2.25	1.6	1.6	1.6	1.85	2.2	2	2.2	2.25	1.8
2.05	2.075	2.025	2.725	2.5	1.8	1.8	1.8	2.05	2.475	2.25	2.475	2.5	2.025
2.25	2.25	2.25	3	2.75	2	2	2	2.25	2.75	2.5	2.75	2.75	2.25

Where(:Principal Component == 2)

Cell Plot

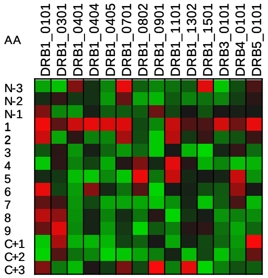

DRB1_0101	DRB1_0301	DRB1_0401	DRB1_0404	DRB1_0405	DRB1_0701	DRB1_0802	DRB1_0901	DRB1_1101	DRB1_1302	DRB1_1501	DRB3_0101	DRB4_0101	DRB5_0101
0.2	0	0.25	0.2	0	0.5	0	0.25	0.4	0	0.4	0	0	0.4
0.3	0.2	0.475	0.32	0.3	0.62	0.25	0.45	0.54	0.2	0.54	0.25	0.2	0.56
0.4	0.4	0.7	0.44	0.6	0.74	0.5	0.65	0.68	0.4	0.68	0.5	0.4	0.72
0.5	0.6	0.925	0.56	0.9	0.86	0.75	0.85	0.82	0.6	0.82	0.75	0.6	0.88
0.6	0.8	1.15	0.68	1.2	0.98	1	1.05	0.96	0.8	0.96	1	0.8	1.04
0.7	1	1.375	0.8	1.5	1.1	1.25	1.25	1.1	1	1.1	1.25	1	1.2
0.8	1.2	1.6	0.92	1.8	1.22	1.5	1.45	1.24	1.2	1.24	1.5	1.2	1.36
0.9	1.4	1.825	1.04	2.1	1.34	1.75	1.65	1.38	1.4	1.38	1.75	1.4	1.52
1	1.6	2.05	1.16	2.4	1.46	2	1.85	1.52	1.6	1.52	2	1.6	1.68
1.1	1.8	2.275	1.28	2.7	1.58	2.25	2.05	1.66	1.8	1.66	2.25	1.8	1.84
1.2	2	2.5	1.4	3	1.7	2.5	2.25	1.8	2	1.8	2.5	2	2

Where(:Principal Component == 3)

Cell Plot

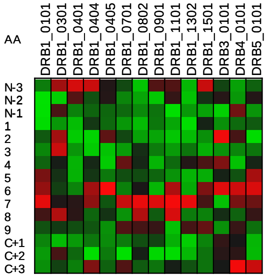

DRB1_0101	DRB1_0301	DRB1_0401	DRB1_0404	DRB1_0405	DRB1_0701	DRB1_0802	DRB1_0901	DRB1_1101	DRB1_1302	DRB1_1501	DRB3_0101	DRB4_0101	DRB5_0101
0.2	0.1	0	0.2	0	0.2	0	0	0.3	0.25	0	0	0.2	0.2
0.34	0.19	0.25	0.34	0.175	0.36	0.25	0.3	0.42	0.5	0.2	0.16	0.32	0.32
0.48	0.28	0.5	0.48	0.35	0.52	0.5	0.6	0.54	0.75	0.4	0.32	0.44	0.44
0.62	0.37	0.75	0.62	0.525	0.68	0.75	0.9	0.66	1	0.6	0.48	0.56	0.56
0.76	0.46	1	0.76	0.7	0.84	1	1.2	0.78	1.25	0.8	0.64	0.68	0.68
0.9	0.55	1.25	0.9	0.875	1	1.25	1.5	0.9	1.5	1	0.8	0.8	0.8
1.04	0.64	1.5	1.04	1.05	1.16	1.5	1.8	1.02	1.75	1.2	0.96	0.92	0.92
1.18	0.73	1.75	1.18	1.225	1.32	1.75	2.1	1.14	2	1.4	1.12	1.04	1.04
1.32	0.82	2	1.32	1.4	1.48	2	2.4	1.26	2.25	1.6	1.28	1.16	1.16
1.46	0.91	2.25	1.46	1.575	1.64	2.25	2.7	1.38	2.5	1.8	1.44	1.28	1.28
1.6	1	2.5	1.6	1.75	1.8	2.5	3	1.5	2.75	2	1.6	1.4	1.4
